# Psychological Impact of the COVID-19 Pandemic on Pregnant Women

**DOI:** 10.3389/fped.2022.790518

**Published:** 2022-04-12

**Authors:** Danilo Buonsenso, Walter Malorni, Arianna Turriziani Colonna, Sofia Morini, Martina Sbarbati, Alessandro Solipaca, Antonio Di Mauro, Brigida Carducci, Antonio Lanzone, Umberto Moscato, Simonetta Costa, Giovanni Vento, Piero Valentini

**Affiliations:** ^1^Department of Woman and Child Health and Public Health, Fondazione Policlinico Universitario A. Gemelli IRCCS, Rome, Italy; ^2^Dipartimento di Scienze Biotecnologiche di Base, Cliniche Intensivologiche e Perioperatorie, Università Cattolica del Sacro Cuore, Rome, Italy; ^3^Global Health Research Institute, Istituto di Igiene, Università Cattolica del Sacro Cuore, Rome, Italy; ^4^National Observatory on Health in the Italian Regions, Istituto di Igiene, Università Cattolica del Sacro Cuore, Rome, Italy; ^5^Pediatric Primary Care, National Pediatric Healthcare System, Margherita di Savoia, Italy

**Keywords:** COVID-19, pregnant women, psychological impact, breastfeeding, newborns

## Abstract

**Objective:**

The aim of this study is to assess the impact of the COVID-19 pandemic on mental health, type of delivery, and neonatal feeding of pregnant women with or without SARS-CoV-2 infection during gestation.

**Study Design:**

The study was conducted online, and anonymous survey was distributed to mothers that delivered during the COVID-19 pandemic.

**Results:**

The survey was completed by 286 women, and 64 women (22.4%) had COVID-19 during pregnancy. Women that had SARS-CoV-2 infection during pregnancy or at time of delivery had a significantly higher probability of being separated from the newborn (*p* < 0.0001) and a significantly lower probability of breastfeeding (*p* < 0.0001). The Edinburg Postnatal Depression Scale, to assess if mothers had symptoms of postnatal depression, showed that items suggestive of postnatal depression were relatively frequent in the whole cohort. However, women with SARS-CoV-2 infection during pregnancy reported higher probability of responses suggestive of postnatal depression in eight out of 10 items, with statistically significant differences in three items.

**Conclusion:**

The COVID-19 pandemic affected the type of delivery and breastfeeding of pregnant women, particularly when they had SARS-CoV-2 infection. This, in turn, had an impact on the psychological status of the interviewed mothers, aspects that could benefit of special support.

## Key Points

- SARS-CoV-2 pregnant women were frequently separated from the newborn and formula fed them.
- SARS-CoV-2 pregnant women more frequently experienced symptoms of postpartum depression.
- The discovery of pregnancy was mainly accompanied by negative feelings, uncertainties, and fears.

## Background

The COVID-19 pandemic has had and is still having devastating global effects, in terms of direct clinical impact (morbidity and mortality), psychological consequences related to drastic changes of daily life, and economic consequences. In this context, the impact on pregnant women has been particularly relevant (1–4).

On a clinical perspective, pregnant women have been susceptible to the SARS-CoV-2, but, differently from the Non-pregnant women of childbearing age, they may have a higher risk of developing symptomatic disease and thromboembolic consequences, given the prothrombotic background of pregnancy itself (1–4). More specifically, the main risks of serious complications from COVID-19 in pregnancy are constituted by the reduced immune response capacity, by the different respiratory dynamics, and finally by the possible greater ease of thromboembolism, which in turn can negatively impact both the obstetric and neonatal outcomes (5). Also, pregnant women faced the related risk of potential clinical consequences of SARS-CoV-2 infection for the fetus (6–8). The natural history of other viral infections [rubella, varicella-zoster virus (9), cytomegalovirus (10), and zika virus (11)] during pregnancy clearly shows that exposed newborns may have a wide range of consequences, from asymptomatic infection to severe disease, congenital malformation, and death (both intrauterine and postnatal). Due to initial uncertainties, almost all national societies opted for a precautionary approach that included mother–newborn separation after delivery and formula feeding in the hypothesis that mothers could transmit the infection postnatally to a susceptible newborn (12). Later on, during the pandemic, original studies and meta-analyses showed that vertical and horizontal mother-to-newborn transmission of SARS-CoV-2 is a rare but possible event (6–8), which most of the time leads to asymptomatic or mild neonatal infection, with long-term outcomes still to explore.

In light of all these uncertainties, becoming infected during pregnancy can have a strong psychological impact on expectant mothers. For this reason, we decided to survey the women who gave birth during the pandemic at our center, in order to understand their psychological burden, and collect data to implement, in future, better management and support for this delicate category of women.

## Materials and Methods

We carried out a cross-sectional survey (Supplementary Material for the full survey). Pregnant women that delivered during the pandemic were asked to fill an online, anonymous Internet-based SurveyMonkey questionnaire (https://it.surveymonkey.com/). Patients were enrolled during a dedicated outpatient service for hip-ultrasound we offer in our institution to every newborn between 6 and 8 weeks of age; therefore, women that delivered at several different institutions received the survey, whose results do not reflect a single center but rather the experience of women from several centers. Most of the mothers delivered the babies at our institution (Fondazione Policlinico Universitario Agostino Gemelli IRCCS in Rome), a place where more than 4,000 children are delivered every year.

We proposed the questionnaire to the mothers of children of this age (6–8 weeks) in order to include the Edinburgh Postnatal Depression Scale (EPDS, available at https://www.fresno.ucsf.edu/pediatrics/downloads/edinburghscale.pdf) in the survey. Participants accessed the survey online using their own computer or mobile device and did not receive a monetary incentive for completion. Individuals without computer or Internet access were not eligible to participate. The data collection took place from October 1, 2020, to January 31, 2021, and contained 38 questions in Italian that were pilot tested with a convenience sample of 25 parents working at the Fondazione Policlinico Universitario A. Gemelli in Rome, Italy. In our center, up until November 2020, mothers were separated from their newborns due to uncertainties around the potential risks of horizontal transmission from the mother to the newborns. When the literature highlighted that the risk of neonatal infection was low (6), we began implementing a dedicated rooming in for mothers with COVID-19, which was implemented since November 2020 and fully working since December 2020. Therefore, almost all interviewed mothers (since they have been interviewed around the third month of infant's life) delivered during a time when separation was still suggested. During this phase, we implemented a social media support channel (mamme&covid) and online meetings to support the families.

The study was approved by the Institute Review Board of our institutions. No personal identifiable data were used.

All variables were summarized as frequencies and percentages. A free distribution test was carried out with the aim of testing the significance of the relationship between psychological impact and infected mother. Specifically, Fisher's exact test was used to overcome the low sample size. All analyses were carried out using Stata software, version 15 (StataCorp, 2017, Stata Statistical Software: Release 15, College Station, Texas, USA: StataCorp LP). To control for type I error related to multiple testing, the significance level was set at *P* = 0.05. The dataset will be made available upon request.

## Results

The survey was completed by 286 women, out of 357 who were invited to participate (80.1% response rate). One hundred and fifty of them (52.4%) had a first child. Two hundred and twenty-two (77.6%) delivered without having SARS-CoV-2 infection, and 64 women (22.4%) had COVID-19 during pregnancy (48% with symptomatic disease).

Overall, 188 women (65.7%) had vaginal delivery, and 168/286 (58.7%) declared that they were without any family member during delivery for reasons related to the COVID-19 pandemic. Similarly, 180 mothers (62.9%) declared they remained alone in the hospital during the postnatal period for local safety rules related with the pandemic.

Women with SARS-CoV-2 infection during pregnancy reported that they were scared for their own health (82.7%), while only 17.3% was not scared, either because they thought that the infection was not severe in young people or because they trusted in the skills of their clinicians.

**Figure 1:**
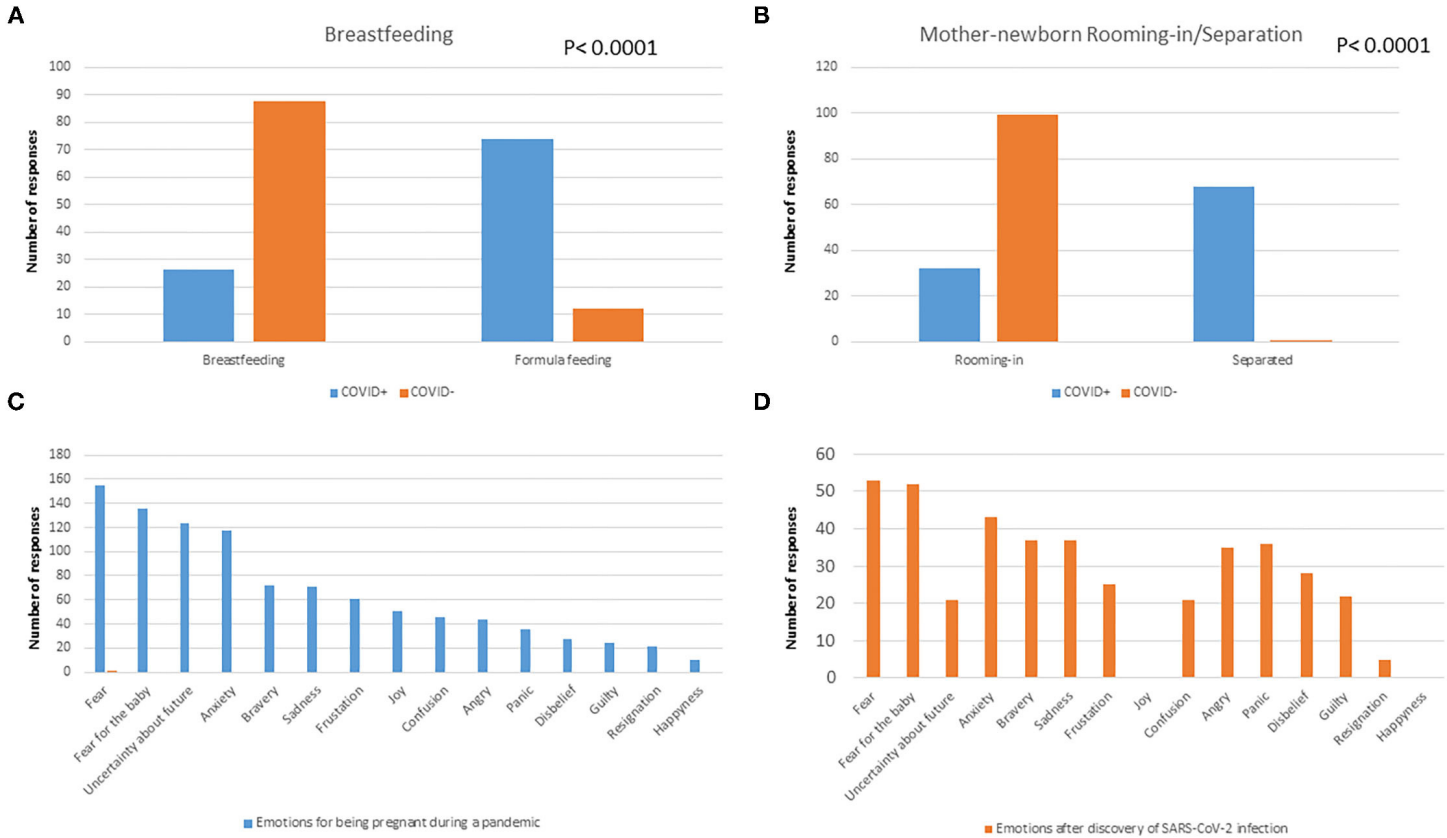
Rate of breastfeeding **(A)** and rooming-in **(B)** according to SARS-CoV-2 infection in pregnancy. The main emotional reactions after women discovered being pregnant **(C)** or having SARS-CoV-2 infection **(D)**.

Women that had SARS-CoV-2 infection during pregnancy or at the time of delivery had a significantly higher probability of being separated from the newborn (*p* < 0.0001, Figure 1A) and a significantly lower probability of breastfeeding (*p* < 0.0001, Figure 1B).

Figures 1C,D shows the mothers' responses when they were asked what they felt when they discovered to be pregnant during the pandemic or after being diagnosed with SARS-CoV-2. Importantly, negative feelings such as fear, fear for the child, uncertainty for the future, anxiety, sadness, and frustration were the most prevalently reported by all mothers. Further details of responses to the survey are reported in the Supplementary Table 1.

The final part of the survey included the Edinburg Postnatal Depression Scale, to assess if mothers had symptoms of postnatal depression and if they were more relevant in women with COVID-19 or those that did not breastfed (Table 1). Overall, items suggestive of postnatal depression were relatively frequent in the whole cohort independent of the “COVID-19” status. However, women with SARS-CoV-2 infection during pregnancy reported more frequent symptoms suggestive of postnatal depression, specifically in the items “have you ever come up with the idea of harming yourself” (*P* = 0.038), “have you been so unhappy that you have cried” (*p* = 0.015), and “have you been looking forward to things with pleasure” (*P* = 0.017). Also, women that did not breastfeed (which happened more frequently in SARS-CoV-2-positive women) reported more frequent pathologic items in the field “have you ever come up with the idea of harming yourself” (*p* = 0.056), “have you been so unhappy that you have cried” (*p* = 0.019), and “have you been able to see the funny side of things” (*p* = 0.02). We have not specifically analyzed the Edinburg Postnatal Depression Scale responses according to whether the mother was separated or not from the newborns, since the separation was significantly associated with the COVID-19 positivity status, and therefore, a significant overlap between the data was expected.

**Table 1:** Edinburg Postnatal Depression Scale results in the whole study population and according to COVID-19 status and breastfeeding.

	**All *N* = 286**	**COVID (+)**	**COVID (–)**	***P*** **value**	**Breastfeeding**	**Formula feeding**	***P*** **value**
**In the past 7 days, have you ever come up with the idea of harming yourself?**							
*Some*	2 (0.7)	**2 (3.13)**	0	**0.034**	0	**2 (2.7)**	**0.056**
*Almost never*	8 (2.8)	3 (4.69)	5 (2.25)		5 (2.36)	3 (4.05)	
*Never*	276 (96.5)	59 (92.19)	217 (97.75)		207 (97.64)	69 (93.24)	
**In the past 7 days, have you been so unhappy that you have cried?**							
*Yes, most of the time*	7 (6.9)	**5 (7.81)**	2 (0.9)	**0.015**	2 (0.94)	**5 (6.76)**	**0.019**
*Yes, quite often*	43 (15)	12 (18.75)	31 (13.96)		28 (13.21)	15 (20.27)	
*Only sometimes*	109 (38.1)	24 (37.5)	85 (38.29)		85 (40.09)	24 (32.43)	
*Never*	127 (45)	23 (35.94)	104 (46.85)		97 (45.75)	30 (40.54)	
**In the past 7 days, have you been feeling sad or disheartened?**							
*Yes, most of the time*	19 (6.6)	5 (7.81)	14 (6.31)	0.689	13 (6.13)	6 (8.11)	0.243
*Yes, quite often*	71 (24.8)	19 (29.69)	52 (23.42)		47 (22.17)	24 (32.43)	
*Only sometimes*	140 (48.9)	29 (45.31)	111 (50)		107 (50.47)	33 (44.59)	
*Never*	56 (19.7)	11 (17.19)	45 (20.27)		45 (21.23)	11 (14.86)	
**In the past 7 days, have you been so unhappy that you have had difficulty sleeping?**							
*Yes, most of the time*	14 (4.9)	**5 (7.81)**	9 (4.05)	0.172	9 (4.25)	5 (6.76)	0.343
*Yes, sometimes*	63 (22)	**18 (28.13)**	45 (20.27)		43 (20.28)	20 (27.03)	
*Not often*	66 (23.1)	10 (15.63)	56 (25.23)		53 (25)	13 (17.57)	
*Never*	143 (50)	31 (48.44)	112 (50.45)		107 (50.47)	36 (48.65)	
**In the past 7 days, have things caused you undue concern?**							
*Yes, most of the time I haven't been able to deal with them*	15 (5.2)	**4 (6.25)**	11 (4.95)	0.676	11 (5.19)	4 (5.41)	0.565
*Yes, sometimes I haven't been able to deal with them as usual*	85 (29.8)	**22 (34.38**)	63 (28.38)		60 (28.30)	25 (33.78)	
*No, most of the time I've handled them pretty well*	137 (47.9)	27 (42.19)	110 (49.55)		101 (48.65)	36 (48.65)	
*No, I dealt with them as well as ever*	49 (17.1**)**	11 (17.19)	38 (17.12)		40 (18.87)	9 (12.16)	
**In the past 7 days, have you been afraid or panicked for no good reason?**							
*Yes, almost always*	18 (6.3)	**6 (9.38)**	12 (5.41)	0.469	11 (5.19)	7 (9.46)	0.282
*Yes, sometimes*	95 (33.2)	**24 (37.5)**	71 (31.98)		74 (34.91)	21 (28.38)	
*No, not often*	73 (25.5)	14 (21.88)	59 (26.58)		57 (26.89)	16 (21.62)	
*Never*	100 (35)	20 (31.25)	80 (36.04)		70 (33.02)	30 (40.54)	
**In the past 7 days, have you been anxious or worried for no good reason?**							
*Yes, often*	44 (15.3)	10 (15.63)	34 (15.32)	1	30 (14.15)	14 (18.92)	0.143
*Yes, sometimes*	123 (42.9)	28 (43.75)	95 (42.79)		99 (46.70)	24 (32.43)	
*Almost never*	60 (20.9)	13 (20.31)	47 (21.17)		40 (18.87)	20 (27.03)	
*No, never*	59 (20.9)	13 (20.31)	46 (20.72)		43 (20.28)	16 (21.62)	
**In the past 7 days, has she unnecessarily blamed herself when things went wrong?**							
*Yes, most of the time*	22 (7.7)	**6 (9.38)**	16 (7.21)	0.545	17 (8.02)	5 (6.76)	0.966
*Yes, sometimes*	78 (27.3)	16 (25)	62 (27.93)		59 (27.83)	19 (25.68)	
*No, not often*	78 (27.3)	14 (21.88)	64 (28.83)		57 (26.89)	21 (28.38)	
*No, never*	108 (37.7)	28 (43.75)	80 (36.04)		79 (37.26)	29 (39.19)	
**In the past 7 days, have you been looking forward to things with pleasure?**							
*As usual*	127 (45)	27 (42.19)	100 (45.45)	**0.017**	95 (45.24)	32 (43.24)	0.675
*A bit less than usual*	81 (28.4)	11 (17.19)	70 (31.82)		62 (29.52)	19 (25.68)	
*Definitely less than usual*	47 (16.5)	**15 (23.44)**	32 (14.55)		34 (16.19)	13 (17.57)	
*Never*	29 (10.1)	**11 (17.19)**	18 (8.18)		19 (9.05)	10 (13.51)	
**In the past 7 days, have you been able to see the funny side of things?**							
*As usual*	88 (30.7)	16 (24.62)	75 (33.78)	0.111	73 (34.43)	18 (24.00)	**0.02**
*A bit less than usual*	74 (25.9)	16 (24.62)	51 (22.97)		49 (23.11)	18 (24.00)	
*Definitely less than usual*	51 (17.9)	9 (13.85)	45 (20.27)		44 (20.75)	10 (13.33)	
*Never*	73 (25.5)	**24 (36.92)**	51 (22.97)		46 (21.70)	**29 (38.67)**	

*The bold values indicate the meaningful data*.

## Discussion

Every pregnancy and every newborn are supposed to be a joyous event in a family. Nevertheless, the results of our survey show a significant change in this picture. The pandemic has affected both psychological and nutritional health of the families. To our knowledge, this is the first survey conducted in Italy to investigate both postpartum depression symptoms and mother–neonate relational aspects in the pandemic age.

Being a mother with COVID-19 appears to be directly related to the increase of depression probability and the need of formula feeding. This evidence identifies the positive new mothers as a new risk category: they may become extremely susceptible to emotional stress and burden; they could feel inadequate toward the child and themselves (“desire of harming themselves”) and deserve special attention. Unfortunately, not only the infected mothers were emotionally affected by the pandemic. Independently from the infection, the pandemic “blue mood” also investigated COVID-free mothers who gave birth in the COVID era. The results confirm what emerged in recent studies about the higher risk and percentage of depression signs in peripartum (13, 14).

In our survey, several mothers declared that they were separated from the newborn due to the infection. Moreover, majority were alone during the peripartum period. The practices of mother–newborn–family separation/inclusion may have been different according to the institution of delivery and the time of the pandemic, since the survey was administered during a long time period and to women that delivered in different centers. In our institution, the management of mother with COVID-19–newborn infant dyads changed during the pandemic: in the initial stages and in the context of the absence of data on rates of perinatal transmission, newborn infants were temporarily separated from their mothers, according to a temporary policy. Subsequently, rooming-in practice was endorsed and encouraged by scientific societies, and our institution was able to guarantee well-separated and safe pathways for health workers within the hospital to manage both pregnant women infected with SARS-CoV-2 and those not infected with SARS-CoV-2, so we reorganized our wards to allow rooming-in.

The mother–newborn separation affects not only the mother but also the baby. The mother–neonate separation leads to a disadvantage in breastfeeding, in favor of formula feeding. In optimal conditions, WHO recommends exclusive breastfeeding in the first 6 months of life (also in June 2020, after the first months of pandemic) (15). Furthermore, evidence exists on the importance of skin-to-skin contact soon after birth, resulting in higher percentage of breastfeeding in neonates once discharged from the hospital (16–18). Breastfeeding is not only a nutritional moment for the mother–infant dyad but is also a relational experience in which all the senses get involved: touch, sight, and smell help building the link between the couple, once the umbilical cord is clamped. The mother learns how to deal with her baby by feeding him, being surrounded by family people. Social isolation, separation from the child (often the first son) soon after the delivery, after 9 months of longing, is hard to cope with, as our results show.

As shown in Table 1, overall, in most of the questions of the Edinburg Postnatal Depression Scale, 20–50% of women provided a response suggestive of a negative mood. Our attempt was not to calculate the overall score of the scale but to analyze the single domains in the overall population and according to COVID-19 or breastfeeding statuses. The finding that in each domain a significant percentage of mothers provided responses suggestive of a negative mood is important, particularly when compared with the Pre-pandemic data available from Italian literature showing that, overall, about 10% of women have scores suggestive of Post-natal depression (19, 20).

There is no doubt that the impact of the pandemic on the mother–newborn relationship was impressive, as much as on the mother's and family's psychological health. Atmuri et al. interviewed 15 Australian women giving birth during the pandemic and underlined how most of them described that “they missed out on a ‘normal' pregnancy experience,” not only for the social restrictions but also for the social distance from the family and especially the partner (21). Kumari et al. also developed a questionnaire to analyze the social impact of COVID-19 on peripartum age (22, 23), suggesting the need to develop strategies to help women dealing with pregnancy in this age. As pediatricians, we focused on the most practical and emotional aspects of the peripartum.

Looking at our results, after months of pregnancies in the COVID era, we can imagine that women and their families could have benefited from psychological support. Being infected with SARS-CoV-2 during gestation and delivery is still a risk factor for physical and mental health, and hospitals should propose support to women who experience this disease and should organize education meetings for healthcare workers to stress the importance of this condition. Unfortunately, the COVID-19 outbreak was so fast and put the health system under so much pressure that the psychological consequences were initially underestimated, and paths of support to patients (and doctors) in that field could not be immediately provided.

Our study has several limitations. First of all, the survey was conducted in Italian. Even though most of the people that access the outpatient clinic of our institution clearly speak and understand Italian, language could have represented a limitation for those foreign parents approaching the survey, who could not complete it. We understand that this is bias in the selection of the population. We can only imagine that foreign families, most of the times belonging to disadvantaged minorities, suffered even more than the Italian families who completed the survey, with language being another barrier during the perinatal period. Another limitation consists in excluding from the survey people who did not have computer or Internet access, but we reasonably believe that this factor was not quantitatively significant in our sample, with the Internet being accessible to almost everyone nowadays. This is only limited to our setting in Rome, during the first wave of the pandemic when Rome was relatively less involved compared to Northern Italy. Therefore, these findings cannot be automatically translated in other settings. However, data from the first wave will allow us to have a comparison group from mothers that delivered during different periods and different practices about separation and feeding. Importantly, we can theoretically expect that these changes in practice may be associated with lower rates of Post-natal depression, since mothers may have had a better perinatal experience.

Although our study is limited by being an online survey self-filled by mothers, it also highlights the important impact of the COVID-19 pandemic on families who were infected or not, especially on the mother–infant dyad, which is the most hit in a very delicate moment and needs special support that is not always promptly provided. Further studies are needed to assess the mid-to-long-term impact of maternal psychological distress on child health.

## Data Availability Statement

The raw data supporting the conclusions of this article will be made available upon request to the corresponding author.

## Ethics Statement

The studies involving human participants were reviewed and approved by Fondazione Policlinico Gemelli of Rome, Italy. The patients/participants provided their written informed consent to participate in this study.

## Author Contributions

DB and PV conceptualized the study. ATC, SM, and MS collected data. AS performed statistical analyses. All authors contributed to the article and approved the submitted version.

## Funding

This study was supported by Mamme&Covid—Surf4Children, a non-profit project partially financed by Fondazione Prosolida, Engie Italia S.p.A, and Terna S.p.A.

## Conflict of Interest

The authors declare that the research was conducted in the absence of any commercial or financial relationships that could be construed as a potential conflict of interest.

## Publisher's Note

All claims expressed in this article are solely those of the authors and do not necessarily represent those of their affiliated organizations, or those of the publisher, the editors and the reviewers. Any product that may be evaluated in this article, or claim that may be made by its manufacturer, is not guaranteed or endorsed by the publisher.
