# Supplementary material for: Psychological Impact of the COVID-19 Pandemic on Pregnant Women
Source: Front Pediatr. 2022 Apr 12;10:790518. doi: 10.3389/fped.2022.790518 (PMC9039297; doi:10.3389/fped.2022.790518)
Supplement: Supplementary file 1 [file Data_Sheet_1.docx]

**Supplementary material**

**FULL SURVEY**

1. Dear Mom, the following anonymous questionnaire is intended to evaluate your maternity experience during the COVID-19 pandemic. We hope that the information obtained from your answers can help us improve the quality of communication with you and your children.
   1. I have read, understood the purpose of the survey and agree to the execution and use of the results for research purposes
2. When was your baby born? Date and time
3. Is it your first child? Yes/No
4. Is it a boy or a girl? Boy/Girl
5. When did you discover COVID-19 positivity?
   1. Days or weeks before giving birth, as I have developed symptoms
   2. Days or weeks before giving birth, as I had close contact with a case of COVID-19
   3. Days or weeks before delivery, as I have tested for other reasons (e.g. screening at work)
   4. I learned about it a few hours before the birth, on the occasion of the screening performed close to the birth
   5. I learned about it shortly after giving birth, on the occasion of the screening performed close to the birth
   6. I've never had COVID-19
   7. I've never had COVID-19 but my partner did
   8. Other (please specify)
6. Mom had a Coronavirus infection:
   1. Asymptomatic
   2. Symptomatic
   3. She has never had COVID-19
7. If symptomatic, what symptoms did the mother have? *(can choose more than one response)*
   1. Fear
   2. Fever or low-grade fever
   3. Headache
   4. Rhinitis
   5. Pneumonia
   6. Muscle aches
   7. Asthenia
   8. Cough
   9. Chills/tremors
   10. Acute breathing difficulty
   11. Inability to perceive odors
   12. Inability to perceive flavors
   13. Burning throat
   14. Difficult breathing
   15. Mental confusion
   16. Diarrhoea
   17. Nausea or vomiting
   18. Loss of appetite
   19. Swollen eyes
   20. Convulsions
   21. Skin rash
   22. The infection was asymptomatic
   23. Other (please specify)
8. If symptomatic, the mother:
   1. She was admitted to the Rooming-in ward
   2. She was admitted to intensive/sub-intensive care
   3. Not applicable
9. The father had a Coronavirus infection:
   1. Asymptomatic
   2. Symptomatic
   3. He has never had COVID-19
10. If symptomatic, what symptoms did dad have? *(can choose more than one response)*
    1. Fear
    2. Fever or low-grade fever
    3. Headache
    4. Rhinitis
    5. Pneumonia
    6. Muscle aches
    7. Asthenia
    8. Cough
    9. Chills/tremors
    10. Acute breathing difficulty
    11. Inability to perceive odors
    12. Inability to perceive flavors
    13. Burning throat
    14. Difficult breathing
    15. Mental confusion
    16. Diarrhoea
    17. Nausea or vomiting
    18. Loss of appetite
    19. Swollen eyes
    20. convulsions
    21. Skin rash
    22. The infection was asymptomatic
    23. Other (please specify)
11. If symptomatic, the father:
    1. He was hospitalized in the normal ward
    2. He was admitted to intensive/sub-intensive care
    3. Other
12. What emotions did you feel when you discovered coronavirus positivity? *(can choose more than one response)*
    1. Fear
    2. Surprise
    3. Uncertainty about the future
    4. Panic
    5. Indifference
    6. Sadness
    7. Joy
    8. Cheerfulness
    9. Need to greet loved ones
    10. Fear of the partner
    11. Fear of the child
    12. Sense of guilt
    13. Anger
    14. Frustration
    15. Disbelief
    16. Resignation
    17. Confusion
    18. Anxiety
    19. Only negative emotions
    20. Only positive emotions
    21. No emotion
    22. We have never had COVID-19
    23. Other (please specify)
13. Were you afraid for your health? *(can choose more than one response)*
    1. Yes, I was afraid that I might develop a severe form of the disease
    2. Yes, but without fearing serious forms of the disease
    3. No, I have always thought that young or relatively young people are at a lower risk of developing severe forms of the disease
    4. No, I knew I was in good hands
    5. I've never had COVID-19
14. How did you feel about expecting a baby during a pandemic? *(can choose more than one response)*
    1. Fear
    2. Surprise
    3. Uncertainty about the future
    4. Panic
    5. Indifference
    6. Sadness
    7. Joy
    8. Cheerfulness
    9. Courage
    10. Fear of the partner
    11. Fear of the child
    12. Sense of guilt
    13. Anger
    14. Frustration
    15. Disbelief
    16. Resignation
    17. Confusion
    18. Anxiety
    19. Only negative emotions
    20. Only positive emotions
    21. No emotions
    22. Other (please specify)
15. If the mother had COVID-19 during pregnancy and close to giving birth, were the parents fearful for the health of the unborn child? *(can choose more than one response)*
    1. Yes, I know that the virus can be transmitted both to the fetus already during pregnancy and to the newborn after delivery, and this can cause symptomatic illness to the newborn, and any long-term effects are still unknown.
    2. Yes, especially due to the uncertainty about the effects it can give to the newborn
    3. No, the virus is not dangerous for the newborn if it becomes infected
    4. No, the virus cannot infect the newborn
    5. Not applicable
    6. Other (please specify)
16. How did the birth take place?
    1. I had a natural birth
    2. I had a caesarean delivery for health issues unrelated to COVID-19
    3. I had a caesarean delivery due to health problems related to the Coronavirus
    4. I had a caesarean delivery by my will, for fear of the Coronavirus
    5. I had a caesarean delivery by my will but not because of the Coronavirus
    6. Other (please specify)
17. During childbirth:
    1. The child's father, or at least another family member, witnessed the delivery
    2. No one, neither the child's father nor another relative of mine, was able to attend the birth for reasons related to COVID-19
18. Have you seen your newborn baby?
    1. I saw it and it was given to me as soon as I was born, allowing me skin-to-skin contact
    2. I saw it without touching it and then I was separated from it, due to COVID-19
    3. I saw it without touching it and then I was separated from it, NOT because of COVID-19 but for other clinical problems
    4. I saw it and I touched it and then I was separated from it, due to COVID-19
    5. I saw and touched it, then I was separated from it, NOT because of COVID-19 but for other clinical problems
    6. I didn't even see it, I was immediately separated from it, due to COVID-19
    7. Other (please specify)
19. After the birth, what was the relationship with the newborn?
    1. We were separated into two different rooms, I never saw him during our stay in the hospital
    2. We were in the same room, but the baby was always in the incubator and at a distance of at least one meter from me, except during breastfeeding
    3. We were in the same room with the baby in a cot, except during breastfeeding
    4. Other (please specify)
20. After the birth, who was the mother with in the hospital?
    1. Alone, no one could be with mom
    2. The child's father, or at least a relative, was in the room with the mother
    3. In a room with another woman with COVID-19
21. During the hospital stay, how was the newborn fed?
    1. He/She took infant formula given by a healthcare professional, as the baby was never in the room with the mother
    2. He/She has taken infant formula given by his mother or father (or in any case by a relative) who wore a mask and always washed his hands
    3. He/She has taken expressed breast milk, given by a doctor, since the baby has never been in the room with his mother.
    4. He/she has taken expressed breast milk, given by a mother or father (or a relative) who wore a face mask and always washed his hands
    5. He was breastfed by his mother, who wore a mask and always sanitized her hands and breasts before breastfeeding
    6. He was breastfed by his mother, who was not wearing a mask
    7. Other (please specify)
22. If the mother was separated from the child, what emotions did she feel about being separated from him/her? *(can choose more than one response)*
    1. Fear
    2. I was not separated from the baby
    3. I was relieved for the sake of the baby
    4. Fear
    5. Surprise
    6. Uncertainty about the future
    7. Panic
    8. Indifference
    9. Sadness
    10. Joy
    11. Cheerfulness
    12. Nostalgia
    13. Impatience
    14. Sense of guilt
    15. Anger
    16. Frustration
    17. Disbelief
    18. Resignation
    19. Confusion
    20. Anxiety
    21. Only negative emotions
    22. Only positive emotions
    23. No emotions
    24. I was not separated from the baby
    25. Other (please specify)
23. Do you think that COVID-19 positivity has affected your ability to be a mother?
    1. Yes
    2. No
    3. I still don't know
    4. Neither my partner nor I have tested positive for COVID-19
    5. Other (please specify)
24. Do you think that COVID-19 positivity has affected your relationship with the child?
    1. Yes
    2. No
    3. I still don't know
    4. Neither my partner nor I have tested positive for COVID-19
    5. Other (please specify)
25. How was the baby breastfed once home?
    1. Breastfed after using a mask and sanitizing the breast before breastfeeding
    2. Breastfed after using a mask without sanitizing the breast before breastfeeding
    3. Breastfeeding without additional COVID-19 related precautions
    4. Mixed breastfeeding (breast milk + formula milk)
    5. Breast milk expressed and given by the father or other relative
    6. Formula feeding only
26. If the baby was separated from the mother in hospital, do you think the separation in the first few days affected the ability to breastfeed the baby?
    1. Yes
    2. No
    3. Don’t know
27. If you have other children, have your brothers/sisters been affected by COVID-19 positivity?
    1. I have no other children
    2. Yes
    3. No
    4. I can't answer
28. If you have other children, did the siblings have any problems with the new baby due to COVID-19 positivity?
    1. I have no other children
    2. Yes
    3. No
    4. I can't answer
29. In the past seven days, have you been able to see the funny side of things?
    1. As usual
    2. A little less than usual
    3. Definitely less than usual
    4. For nothing
30. In the past seven days, have you been looking forward to things with pleasure?
    1. As usual
    2. A little less than usual
    3. Definitely less than usual
    4. For nothing
31. In the past seven days, have you unnecessarily blamed yourself when things went wrong?
    1. Yes, most of the time
    2. Yes, sometimes
    3. Not very often
    4. Never
32. In the past seven days, have you been anxious or worried for no good reason?
    1. No, not at all
    2. Almost never
    3. Yes, sometimes
    4. Yes, often
33. In the past seven days, have you been afraid or panicked for no good reason?
    1. Yes, almost always
    2. Yes, sometimes
    3. No, not very often
    4. Never
34. In the past seven days, have things caused you undue concern?
    1. Yes, most of the time I haven't been able to deal with them
    2. Yes, sometimes I haven't been able to deal with them as usual
    3. No, most of the time I've handled them pretty well
    4. No, I dealt with them as well as ever
35. In the past seven days, have you been so unhappy that you have had difficulty sleeping?
    1. Yes, most of the time
    2. Yes, sometimes
    3. Not very often
    4. No, not at all
36. In the past seven days, did you feel sad or disheartened?
    1. Yes, most of the time
    2. Yes, quite often
    3. Only occasionally
    4. Never
37. In the past seven days, have you been so unhappy that you have plans?
    1. Yes, most of the time
    2. Yes, quite often
    3. Only occasionally
    4. Never
38. In the past seven days, have you ever come up with the idea of ​​harming yourself?
    1. Yes, quite often
    2. Sometimes
    3. Almost never
    4. Never

**Supplementary Table S1.** Responses to the survey

| **3. Is it your first child?** | **N** | **(%)** |
| --- | --- | --- |
| a. Yes | 150 | 52,45 |
| b. No | 136 | 47,55 |
| **4. Is it a boy or a girl?** | **N** | **(%)** |
| a. Boy | 139 | 48,6 |
| b. Girl | 147 | 51,4 |
| **5. When did you discover COVID-19 positivity?** | **N** | **(%)** |
| a. Days or weeks before giving birth, as I have developed symptoms | 19 | 6,64 |
| b. Days or weeks before giving birth, as I had close contact with a case of COVID-19 | 13 | 4,55 |
| c. Days or weeks before delivery, as I have tested for other reasons (e.g. screening at work) | 9 | 3,15 |
| d. I learned about it a few hours before the birth, on the occasion of the screening performed close to the birth | 17 | 5,94 |
| e. I learned about it shortly after giving birth, on the occasion of the screening performed close to the birth | 7 | 2,45 |
| f. I've never had COVID-19 | 206 | 72,02 |
| g. I've never had COVID-19 but my partner did | 4 | 1,4 |
| h. Other (please specify) | 11 | 3,85 |
| **6. Mom had a Coronavirus infection:** | **N** | **(%)** |
| a.      Asymptomatic | 35 | 11,89 |
| b.      Symptomatic | 39 | 13,63 |
| c.      She has never had COVID-19 | 212 | 74,48 |
| **7. If symptomatic, what symptoms did the mother have?** | **N** | **(%)** |
| a.      Fever or low-grade fever | 30 | 26,55 |
| b.      Headache | 15 | 13,27 |
| c.      Rhinitis | 15 | 13,27 |
| d.      Pneumonia | 9 | 7,96 |
| e.      Muscle aches | 18 | 15,93 |
| f.       Asthenia | 9 | 7,96 |
| g.      Cough | 16 | 14,16 |
| h.      Chills/tremors | 17 | 15,04 |
| i.       Acute breathing difficulty | 5 | 4,42 |
| j.       Inability to perceive odors | 31 | 27,43 |
| k.      Inability to perceive flavors | 32 | 28,32 |
| l.       Burning throat | 5 | 4,42 |
| m.    Difficult breathing | 8 | 7,08 |
| n.      Mental confusion | 4 | 3,54 |
| o.      Diarrhoea | 10 | 8,85 |
| p.      Nausea or vomiting | 2 | 1,77 |
| q.      Loss of appetite | 13 | 11,5 |
| r.       Swollen eyes | 1 | 0,88 |
| s.      Convulsions | 0 | 0 |
| t.       Skin rash | 1 | 0,88 |
| u.      The infection was asymptomatic | 5 | 4,42 |
| v.      Other (please specify) | 67 | 59,29 |
| **8. If symptomatic, the mother:** | **N** | **(%)** |
| a.      She was admitted to the Rooming-in ward | 16 | 25,00 |
| b.      She was admitted to intensive/sub-intensive care | 6 | 9,33 |
| **9. The father had a Coronavirus infection:** | **N** | **(%)** |
| a.      Asymptomatic | 19 | 6,7 |
| b.      Symptomatic | 36 | 12,6 |
| c.      He has never had COVID-19 | 231 | 80,7 |
| **10. If symptomatic, what symptoms did dad have?** | **N** | **(%)** |
| a.      Fever or low-grade fever | 28 | 24,56 |
| b.      Headache | 16 | 14,04 |
| c.      Rhinitis | 8 | 7,02 |
| d.      Pneumonia | 1 | 0,88 |
| e.      Muscle aches | 22 | 19,3 |
| f.       Asthenia | 5 | 4,39 |
| g.      Cough | 20 | 17,54 |
| h.      Chills/tremors | 9 | 7,89 |
| i.       Acute breathing difficulty | 2 | 1,75 |
| j.       Inability to perceive odors | 23 | 20,18 |
| k.      Inability to perceive flavors | 25 | 21,93 |
| l.       Burning throat | 5 | 4,39 |
| m.    Difficult breathing | 3 | 2,63 |
| n.      Mental confusion | 0 | 0 |
| o.      Diarrhoea | 2 | 1,75 |
| p.      Nausea or vomiting | 1 | 0,88 |
| q.      Loss of appetite | 4 | 3,51 |
| r.       Swollen eyes | 4 | 3,51 |
| s.      convulsions | 1 | 0,88 |
| t.       Skin rash | 0 | 0 |
| u.      The infection was asymptomatic | 7 | 6,14 |
| v.      Other (please specify) | 64 | 56,14 |
| **11. If symptomatic, the father:** | **N** | **(%)** |
| a.      He was admitted to the Rooming-in ward | 1 | 0,3 |
| b.      He was admitted to intensive/sub-intensive care | 0 | 0 |
| c.      Other | 285 | 99,7 |
| **12. What emotions did you feel when you discovered coronavirus positivity?** | **N** | **(%)** |
| a.      Fear | 53 | 2,08 |
| b.      Surprise | 17 | 7,08 |
| c.      Uncertainty about the future | 21 | 8,75 |
| d.      Panic | 36 | 15 |
| e.      Indifference | 2 | 0,83 |
| f.       Sadness | 37 | 15,42 |
| g.      Joy | 0 | 0 |
| h.      Cheerfulness | 0 | 0 |
| i.       Need to greet loved ones | 5 | 2,08 |
| j.       Fear of the partner | 21 | 8,75 |
| k.      Fear of the child | 52 | 21,67 |
| l.       Sense of guilt | 22 | 9,17 |
| m.    Anger | 35 | 14,58 |
| n.      Frustration | 25 | 10,42 |
| o.      Disbelief | 28 | 11,67 |
| p.      Resignation | 5 | 2,08 |
| q.      Confusion | 21 | 8,75 |
| r.       Anxiety | 43 | 17,92 |
| s.      Only negative emotions | 24 | 10 |
| t.       Only positive emotions | 0 | 0 |
| u.      No emotion | 1 | 0,42 |
| v.      We have never had COVID-19 | 162 | 57,5 |
| w.    Other (please specify) | 3 | 1,25 |
| **13. Were you afraid for your health?** | **N** | **(%)** |
| a.      Yes, I was afraid that I might develop a severe form of the disease | 42 | 14,6 |
| b.      Yes, but without fearing serious forms of the disease | 25 | 8,7 |
| c.      No, I have always thought that young or relatively young people are at a lower risk of developing severe forms of the disease | 9 | 3,2 |
| d.      No, I knew I was in good hands | 6 | 2,2 |
| e.      no applicable | 204 | 71,3 |
| **14. How did you feel about expecting a baby during a pandemic?** | **N** | **(%)** |
| a.      Fear | 155 | 55,36 |
| b.      Surprise | 4 | 1,43 |
| c.      Uncertainty about the future | 124 | 44,29 |
| d.      Panic | 35 | 12,5 |
| e.      Indifference | 1 | 0,36 |
| f.       Sadness | 71 | 25,36 |
| g.      Joy | 51 | 18,21 |
| h.      Cheerfulness | 10 | 3,57 |
| i.       Courage | 72 | 25,71 |
| j.       Fear of the partner | 22 | 7,86 |
| k.      Fear of the child | 136 | 48,57 |
| l.       Sense of guilt | 24 | 8,57 |
| m.    Anger | 44 | 15,71 |
| n.      Frustration | 61 | 21,79 |
| o.      Disbelief | 27 | 9,64 |
| p.      Resignation | 21 | 7,5 |
| q.      Confusion | 46 | 16,43 |
| r.       Anxiety | 117 | 41,79 |
| s.      Only negative emotions | 12 | 4,29 |
| t.       Only positive emotions | 13 | 4,64 |
| u.      No emotions | 1 | 0,36 |
| v.      Other (please specify) | 10 | 3,57 |
| **15. If the mother had COVID-19 during pregnancy and close to giving birth, were the parents fearful for the health of the unborn child?** | **N** | **(%)** |
| a.      Yes, I know that the virus can be transmitted both to the fetus already during pregnancy and to the newborn after delivery, and this can cause symptomatic illness to the newborn, and any long-term effects are still unknown. | 20 | 7,76 |
| b.      Yes, especially due to the uncertainty about the effects it can give to the newborn | 40 | 15,5 |
| c.      No, the virus is not dangerous for the newborn if it becomes infected | 4 | 1,55 |
| d.      No, the virus cannot infect the newborn | 4 | 1,55 |
| e.      Not applicable | 187 | 72,48 |
| f.       Other (please specify) | 3 | 1,16 |
| **16. How did the birth take place?** | **N** | **(%)** |
| a.      I had a natural birth | 188 | 65,7 |
| b.      I had a caesarean delivery for health issues unrelated to COVID-19 | 56 | 19,6 |
| c.      I had a caesarean delivery due to health problems related to the Coronavirus | 2 | 0,7 |
| d.      I had a caesarean delivery by my will, for fear of the Coronavirus | 2 | 0,7 |
| e.      I had a caesarean delivery by my will but not because of the Coronavirus | 13 | 4,6 |
| f.       Other (please specify) | 25 | 8,7 |
| **17. During childbirth:** | **N** | **(%)** |
| a.      The child's father, or at least another family member, witnessed the delivery | 109 | 38,4 |
| b.      No one, neither the child's father nor another relative of mine, was able to attend the birth for reasons related to COVID-19 | 177 | 61,6 |
| **18. Have you seen your newborn baby?** | **N** | **(%)** |
| a.      I saw it and it was given to me as soon as I was born, allowing me skin-to skin contact | 175 | 61,2 |
| b.      I saw it without touching it and then I was separated from it, due to COVID-19 | 30 | 10,5 |
| c.      I saw it without touching it and then I was separated from it, NOT because of COVID-19 but for other clinical problems | 15 | 5,2 |
| d.      I saw it and I touched it and then I was separated from it, due to COVID19 | 18 | 6,3 |
| e.      I saw and touched it, then I was separated from it, NOT because of COVID-19 but for other clinical problems | 14 | 4,9 |
| f.       I didn't even see it, I was immediately separated from it, due to COVID-19 | 4 | 1,4 |
| g.      Other (please specify) | 30 | 10,5 |
| **19. After the birth, what was the relationship with the newborn?** | **N** | **(%)** |
| a.      We were separated into two different rooms, I never saw him during our stay in the hospital | 45 | 15,7 |
| b.      We were in the same room, but the baby was always in the incubator and at a distance of at least one meter from me, except during breastfeeding | 6 | 2,1 |
| c.      We were in the same room with the baby in a cot, except during breastfeeding | 122 | 42,6 |
| d.      Other (please specify) | 113 | 39,6 |
| **20. After the birth, who was the mother with in the hospital?** | **N** | **(%)** |
| a.      Alone, no one could be with mom | 180 | 62,9 |
| b.      The child's father, or at least a relative, was in the room with the mother | 62 | 21,6 |
| c.      In a room with another woman with COVID-19 | 33 | 11,6 |
| d.      Other | 11 | 3,9 |
| **21. During the hospital stay, how was the newborn fed?** | **N** | **(%)** |
| a.      He/She took infant formula given by a healthcare professional, as the baby was never in the room with the mother | 47 | 16,79 |
| b.      He/She has taken infant formula given by his mother or father (or in any case by a relative) who wore a mask and always washed his hands | 3 | 1,07 |
| c.      He/She has taken expressed breast milk, given by a doctor, since the baby has never been in the room with his mother. | 3 | 1,07 |
| d.      He/she has taken expressed breast milk, given by a mother or father (or a relative) who wore a face mask and always washed his hands | 2 | 0,71 |
| e.      He was breastfed by his mother, who wore a mask and always sanitized her hands and breasts before breastfeeding | 60 | 20,43 |
| f.       He was breastfed by his mother, who was not wearing a mask | 152 | 53,29 |
| g.      Other (please specify) | 19 | 6,64 |
| **22. If the mother was separated from the child, what emotions did she feel about being separated from him/her?** | **N** | **(%)** |
| a.      I was not separated from the baby | 108 | 44,08 |
| b.      I was relieved for the sake of the baby | 6 | 2,45 |
| c.      Fear | 20 | 8,16 |
| d.      Surprise | 1 | 0,41 |
| e.      Uncertainty about the future | 12 | 4,9 |
| f.       Panic | 15 | 6,12 |
| g.      Indifference | 1 | 0,41 |
| h.      Sadness | 50 | 20,41 |
| i.       Joy | 1 | 0,41 |
| j.       Cheerfulness | 0 | 0 |
| k.      Nostalgia | 23 | 9,39 |
| l.       Impatience | 28 | 11,43 |
| m.    Sense of guilt | 21 | 8,57 |
| n.      Anger | 29 | 11,84 |
| o.      Frustration | 30 | 12,24 |
| p.      Disbelief | 9 | 3,67 |
| q.      Resignation | 9 | 3,67 |
| r.       Confusion | 16 | 6,53 |
| s.      Anxiety | 23 | 9,39 |
| t.       Only negative emotions | 18 | 7,35 |
| u.      Only positive emotions | 0 | 0 |
| v.      No emotions | 0 | 0 |
| w.    I was not separated from the baby | 77 | 31,43 |
| x.      Other (please specify) | 4 | 1,63 |
| **23. Do you think that COVID-19 positivity has affected your ability to be a mother?** | **N** | **(%)** |
| a.      Yes | 13 | 20,3 |
| **24. Do you think that COVID-19 positivity has affected your relationship with the child?** | **N** | **(%)** |
| a.      Yes | 29 | 45,3 |
| **25. How was the baby breastfed once home?** | **N** | **(%)** |
| a.      Breastfed after using a mask and sanitizing the breast before breastfeeding | 31 | 10,8 |
| b.      Breastfed after using a mask without sanitizing the breast before breastfeeding | 21 | 7,3 |
| c.      Breastfeeding without additional COVID-19 related precautions | 149 | 52,1 |
| d.      Mixed breastfeeding (breast milk + formula milk) | 61 | 21,3 |
| e.      Breast milk expressed and given by the father or other relative | 3 | 1 |
| f.       Formula feeding only | 14 | 5,1 |
| g.       Other | 7 | 2,4 |
| **26. If the baby was separated from the mother in hospital, do you think the separation in the first few days affected the ability to breastfeed the baby?** | **N** | **(%)** |
| a.      Yes | 44 | 15,4 |
| b.      No | 20 | 6,9 |
| c.      Don’t know | 222 | 77,7 |
| **27. If you have other children, have your brothers/sisters been affected by COVID-19 positivity?** | **N** | **(%)** |
| a.      I have no other children | 96 | 33,5 |
| b.      Yes | 23 | 8 |
| c.      No | 11 | 4 |
| d.      I can't answer | 156 | 54,5 |
| **28. If you have other children, did the siblings have any problems with the new baby due to COVID-19 positivity?** | **N** | **(%)** |
| a.      I have no other children | 101 | 35,3 |
| b.      Yes | 7 | 2,4 |
| c.      No | 32 | 11,2 |
| d.      I can't answer | 146 | 51,1 |
